# Electrochemical Deposition and Nucleation/Growth Mechanism of Ni–Co–Y_2_O_3_ Multiple Coatings

**DOI:** 10.3390/ma11071124

**Published:** 2018-07-01

**Authors:** Xinyu Zhou, Yiyong Wang, Zhipeng Liang, Hui Jin

**Affiliations:** School of Materials & Metallurgy, University of Science and Technology Liaoning, Anshan 114051, China; zxyustl@163.com (X.Z.); zplustl@163.com (Z.L.); hui313@163.com (H.J.)

**Keywords:** Ni–Co–Y_2_O_3_ composite, chronoamperometry, instantaneous nucleation, kinetic parameters

## Abstract

Ni–Co alloy and Ni–Co–Y_2_O_3_ multiple coatings refined with nano-Y_2_O_3_ particles were fabricated by ultrasonic-assisted electrochemical deposition in an acid sulfamate bath. Linear sweep voltammetry (LSV), chronoamperometry (CA) and electrochemical impedance spectroscopy (EIS) techniques were applied to investigate the nucleation/growth process of composite coatings in co-deposition. The LSV results indicated that the incorporation of nano-Y_2_O_3_ particles with the Ni–Co matrix shifted the initial deposition potential to a more positive potential and decreased cathodic polarization. For both coatings, the nucleation/growth process approximately agreed with the Scharifker–Hill instantaneous nucleation model. Nucleation parameters, including active nucleation sites (N_0_) and nucleation rate (A) of the composite, were higher when the measured potential range was between −1.05 V (vs. SCE) and −1.20 V vs. SCE, when compared with the Ni–Co alloy, and the observed AFM images of the coatings were in good agreement with the calculated nucleation parameters (using the Marquardt–Levenberg algorithm) of experimental curves. EIS testing indicated that the charge transfer resistance of the composite was lower in electrodeposition. The incorporation of nano-Y_2_O_3_ particles in the matrix changed the preferred orientation of coatings and produced a more uniform and compact deposit layer with finer grains.

## 1. Introduction

Composite plating is a technology using the electrochemical deposition technique to make solid particles (Al_2_O_3_, SiC, ZrO_2_, WC, SiO_2_, BN, Cr_2_O_3_, Si_3_N_4_, B_4_C, and others) embed in a metal matrix (e.g., Ni, Cu, Ag, Co, Cr) to obtain special composite coatings [1,2]. The electrochemical deposited composite coatings collectively contain unique functions from both solid particles and the metal matrix. Electrodeposition is a convenient and effective method of preparing multifunctional compound coatings and, according to the design requirements, the physical and chemical performances of composite coatings can be regulated by adjusting the electrochemical deposition parameters [3]. This leads to a new development of composite coatings widely used in many engineering fields owing to their outstanding characteristics. Consequently, there has recently been a rapidly increasing interest in metal/particle composite coatings fabricated by electrochemical deposition [4]. Ni–Co coating has attracted great attention owing to its outstanding functions including high hardness, corrosion resistance, and thermal stability [5,6,7] making it suitable to be applied as a metallic matrix. Furthermore, to achieve superior performance, through electrodeposition techniques, various particles (SiC [8,9], Al_2_O_3_ [10], Cr_2_O_3_ [11], CNT [12], among others) have been embedded in the Ni–Co matrix. Yttrium oxide (Y_2_O_3_) has been widely applied in materials and chemical engineering owing to its unique physical and chemical properties including high melting temperature, transparency, and high corrosion resistance [13]. In fact, it is reported that Ni–Y_2_O_3_ composite coatings have been fabricated under direct current deposition, and the microhardness, wear, and corrosion resistance of composite coatings are significantly improved through the incorporation of nano-Y_2_O_3_ particles in a nickel matrix [14,15]. Accordingly, it would be expected that the co-deposition between a Ni–Co matrix and nano-Y_2_O_3_ particles may allow the obtention of a superior performance. 

The electrodeposition of composite coatings is influenced by several operating parameters, such as the composition of the plating bath, particle characteristics, applied cathodic current density, solution temperature, and pH [16]. At the same time, the electro-crystallization process of the fresh layer on a substrate surface is significant to the further growth and performance of coatings. Ordinarily, an electro-crystallization process can be divided to two stages: nucleation and growth [17,18]. The competition between nucleation and growth determines the size and morphology of the sediments, and then affects the properties of the coating [19]. There have been many articles reporting the electro-crystallization process of metal coatings. Tebbakh et al. [20] illustrated that the electro-crystallization process of Co and Ni–Co alloys in the chlorinated system follows a 3D instantaneous nucleation/growth model controlled by diffusion. Tan et al. [21] reported that the nucleation/growth of an Ni–SiC composite coating in a Watt-type plating solution conforms to a progressive nucleation mechanism at low overpotential. Contrarily, it follows the instantaneous nucleation mechanism at high overpotential. Ghaziof et al. [19] determined that the nucleation of Zn–Ni alloy in the presence of nano-Al_2_O_3_ particles shifts from a progressive mechanism to an instantaneous mechanism, and the number of active nucleation sites and the nucleation rate significantly increase. Accordingly, the adsorption of particles on the electrode surface has a critical influence on the nucleation/growth of deposits. There have been numerous theoretical models used to explain the co-deposition mechanism of metal-particle composite coatings, and in particular Guglielmi’s two-step adsorption model [22], which is able to treat the influences of particles on the depositing rate of composite coating. 

The aim of this research was to study the electrochemical behavior of nano-Y_2_O_3_ particles in co-deposition through LSV curves. CA curves were measured to probe the influence of nano-Y_2_O_3_ particles on the nucleation rate. Fit calculations were applied to the CA curves to obtain electrochemical deposition parameters. Atomic force microscopy (AFM) was used to offer a favorable reference for the calculated nucleation rate in electrodeposition. The surface morphology and microstructure of the composite coatings were characterized by scanning electron microscopy (SEM) and X-ray diffractometer (XRD).

## 2. Materials and Methods 

### 2.1. Materials

**Figure 1:**
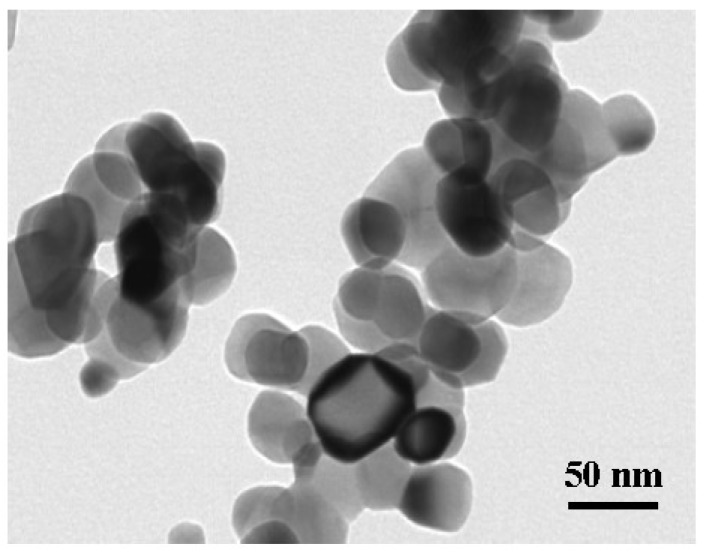
TEM image of nano-Y_2_O_3_ particles.

Ni–Co–Y_2_O_3_ composite coatings were electrodeposited from a standard sulfamate aqueous electrolytic solution. The plating bath was composed of distilled water and analytical grade pure chemicals: Ni(NH_2_SO_3_)_2_·4H_2_O 80 g/L, Co(NH_2_SO_3_)_2_·4H_2_O 16 g/L, H_3_BO_3_ 40 g/L, and nano-Y_2_O_3_ particles 10 g/L (average grain diameter around 50 nm, in Figure 1, measured by transmission electron microscope (TEM), JEM-2100). A pure nickel sheet (99.99%) was used as an anode, and a pure copper plate (99.99%) as a cathode, with a surface of 1 cm^2^. Before electrodeposition, the working face was polished with different grit emery papers (400, 800, 1200), then washed in distilled water, and activated in 5% HCl solution for 10 s. A high-frequency direct current power (model PS-618) was used to apply current density at 3 A/dm^2^ in electrodeposition. The temperature was kept at 40 ± 2 °C, and the pH was 4 ± 0.2 in a plating bath of 300 mL. An intelligent controlled temperature ultrasonic synthetic extractor (XH-2008DE model, Xianghu, Beijing, China) was used to maintain the temperature and offer an ultrasonic power of 100 W with 35 kHz in deposition.

### 2.2. Methods

The electrochemical experiments were measured through a traditional three-electrode system in conditions coincident with the preparation of coatings, where a copper plate was the working electrode (WE), a platinum plate was the counter electrode (CE), and a saturated calomel electrode was the reference electrode (SCE). The LSV experiments were carried out at potential ranging from 0 V down to −2.0 V, at a scan rate −30 mV/s. The CA curves were measured from −1.05 V to −1.20 V, the recorded time was 120 s. EIS experiments were carried out at frequencies of 10^−1^ to 10^5^ Hz at different deposition potentials. An autolab electrochemical workstation (AUT85731, Nova1.9, Metrohm, Switzerland) was used for electrochemical measurements. The X-ray diffraction technique (X’Pert Powder, PANalytical, Almelo, The Netherlands) was used to study the average grain size and the preferred orientation of deposits (Cu Kα filtered radiation, step = 0.02°, scanning speed = 10°/min, 2 theta ranged from 10° to 90°). The surface morphology of the coatings was complemented by SEM (1 KV~15 KV, Zeiss-ΣIGMA HD, Carl Zeiss, Oberkochen, Germany). The atomic percentage of each element in the deposits was investigated by EDS in SEM equipped with an OXFORD-X-Max 50 mm^2^ spectrometer (Oxford Instruments, Abingdon, UK). The morphology of both coatings fabricated for different electrodeposition time was characterized by AFM (CSPM5500, Guangzhou, China), and the scanning area was 100 μm^2^.

## 3. Results and Discussion 

### 3.1. Linear Sweep Voltammetry (LSV)

**Figure 2:**
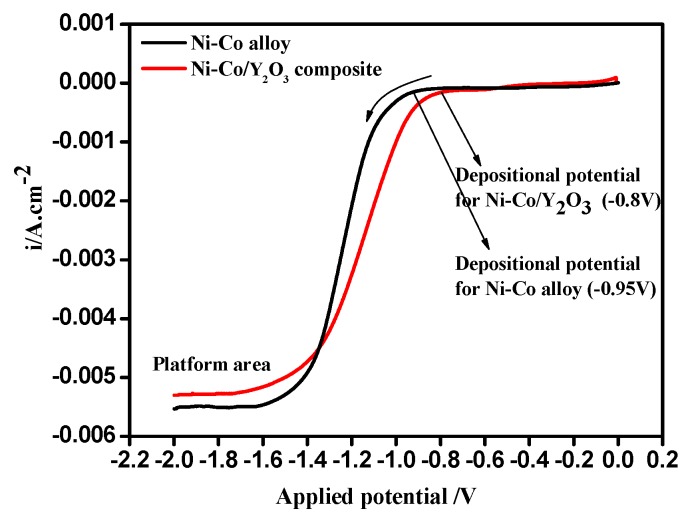
Linear sweep voltammetry curves on pure copper in electrolyte with and without nano-Y_2_O_3_ particles, ultrasonic power 100 W, T = 40 °C, and pH = 4.

The linear sweep voltammetry curves measured on the copper surface in the sulfamate electrolyte solution with and without nano-Y_2_O_3_ particles are shown in Figure 2, which was measured at potential ranging from 0 V down to −2.0 V, and with a scan rate of −30 mV/s. 

**Figure 3:**
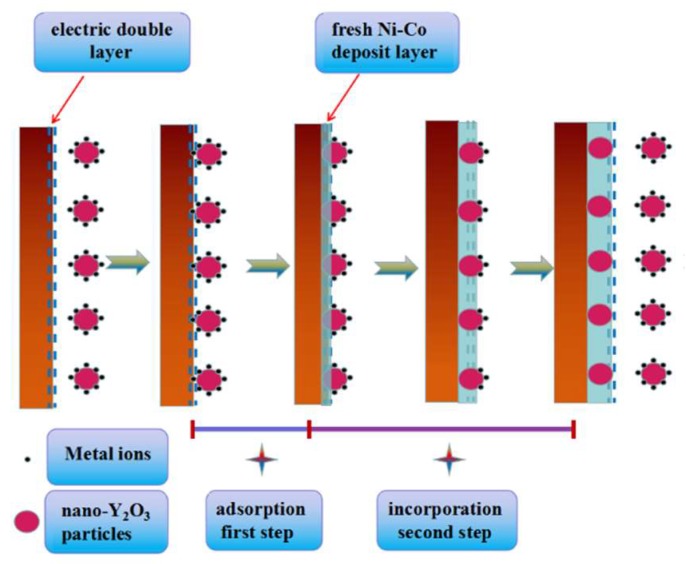
Schematic of the electrochemical co-deposition process between metal ions and nano-Y_2_O_3_ particles on the pure copper.

As shown in Figure 2, the cathodic current density of the Ni–Co alloy clearly increases at −0.95 V (vs. SCE), which shows that the reduction of Ni^2+^ and Co^2+^ takes place; however, the reduction potential of Ni–Co–Y_2_O_3_ composite is about at −0.80 V (vs. SCE). The addition of nano-Y_2_O_3_ particles to the electrolyte shifts the electrodeposition overpotential of the Ni–Co deposit to a more positive value, which results in a decrease of cathodic polarization. This can be attributed to a possible conformation change of the electric double layer owing to the adsorption of particles on the cathode surface [21]. Furthermore, compared to the Ni–Co alloy, the current density of the composite is higher when the potential is greater than −1.20 V (vs. SCE), but the cathodic current density of the Ni–Co–Y_2_O_3_ composite coating is lower at a potential range from −1.20 V to −2.0 V (vs. SCE). This difference may be related to the adsorption strength of nano-Y_2_O_3_ particles at different potentials, which can be illustrated by Guglielmi’s two-step adsorption model [22] (see Figure 3). According to Gugliemi’s absorption model [22], the electrochemical deposition process between metal ions and nano-Y_2_O_3_ particles can be divided into two steps: firstly, the nano-Y_2_O_3_ particles are weakly adsorbed on the matrix surface under low electrical field forces when the applied potential is greater than −1.20 V (vs. SCE), and as the weakly absorbed particles extended the active action sites on electrode surface, the number of reduced ions increased, resulting in an increase of cathodic current density. Secondly, the nano-Y_2_O_3_ particles were embedded in a fresh Ni–Co matrix and consequently formed composites; the incorporated nano-Y_2_O_3_ particles (non-conductor) covered the active reaction sites and showed a significant spatial hindrance effect, and the number of reduced ions decreased, resulting in a decrease of cathodic current density. In this potential range, a platform area appeared, the electrode surface was controlled by diffusion, and the current density was closer to a limited diffusion current density.

**Figure 4:**
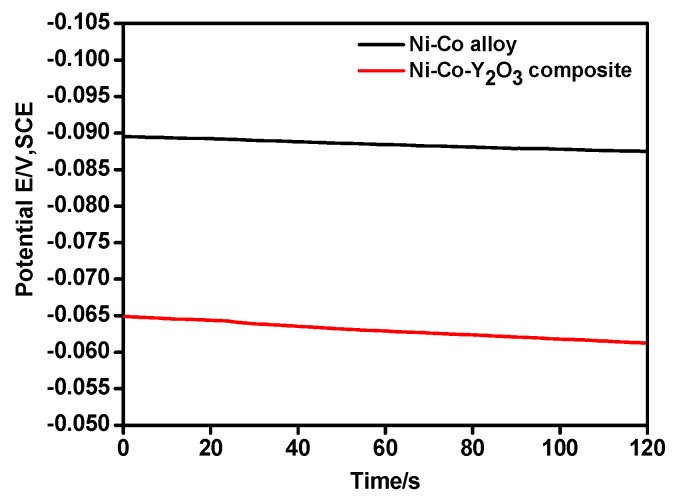
Open circuit potential curves of Ni–Co alloy and Ni–Co–Y_2_O_3_ composite.

Figure 4 shows the open circuit potential (OCP) of Ni–Co and Ni–Co–Y_2_O_3_ depositing in the electrolyte. The OCP of the composite coating was more positive than the alloy, indicating that less energy was required for the composite electrodeposition. Accordingly, the cathodic current density measured in the composite electrolyte was higher than that recorded in the Ni^2+^ and Co^2+^ electrolyte at the initial electrodeposition stage, a result consistent with Figure 2. 

### 3.2. Chronoamperometry Study

**Figure 5:**
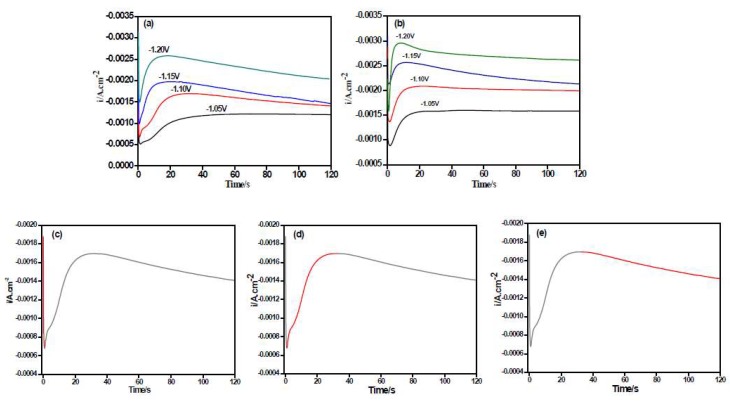
I~t curves of co-deposition at different step potential on copper electrode: (**a**) Ni–Co alloy; (**b**) Ni–Co–Y_2_O_3_ composite; (**c**) depicted stages are prior to electroreduction; (**d**) state at the onset of reduction; and (**e**) steady state of electroreduction.

Chronoamperometry is an effective way to study the nucleation/growth model of metals in the electro-crystallization process [23]. The I~t transient curves which measured the deposition of the Ni–Co alloy and the Ni–Co–Y_2_O_3_ composite coatings in the sulfamate electrolyte bath are shown in Figure 5a,b, respectively. The measured step potential range was from −1.05 V to −1.20 V (vs. SCE), the corresponding time was 120 s, the temperature was 25 °C, and the pH was 4.

In both Figure 5a,b, the I~t curves have a quintessential diffusion-controlled 3D nucleation feature [23], and the I~t curves can be divided into three parts (Figure 5c–e). The first part is near the longitudinal axis for the decline of current density, which corresponds to the charge of the electric double layer on the electrode surface. In the second part, the current density gradually increased and reached the maximum value, which was a typical crystal nucleation/growth process; the cathodic current density gradually decreased because of the diffusion of deposits from the electrolyte to the electrode/solution interface in the third part [23,24]. In I~t curves, it is noteworthy that the maximum current density (*I_m_*) gradually rises by making the potential more negative, while the corresponding time (*t_m_*) decreases, which indicates an increase in the nucleation rate. This may be because the higher electric field force under more negative potential increases the active nucleation sites on the electrode surface, promoting the electro-crystallization process. Moreover, at measured potentials, the maximum value of the current density (*I_m_*) for Ni–Co–Y_2_O_3_ composites is higher than that in the Ni–Co curves, while the corresponding relaxation time (*t_m_*) is lower, as seen in Table 1. 

**Figure 6:**
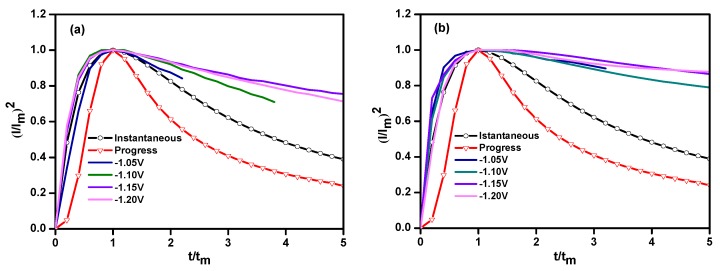
Dimensionless curves of the composite co-deposition process on the copper electrode: (**a**) Ni–Co alloy; (**b**) Ni–Co–Y_2_O_3_ composites, ultrasonic power 100 W, T = 40 °C, and pH = 4.

To characterize the nucleation process of both coatings, the data of the CA curves were normalized to (*I/I_m_*)^2^~(*t/t_m_*) curves and compared with the Scharifker–Hills model [23] based on Equations (1) and (2). The obtained non-dimensional (*I/I_m_*)^2^~(*t/t_m_*) curves are shown in Figure 6.

(1) $${\left(I/I_{m}\right)}^{2}=\frac{1.9542}{\left(t/t_{m}\right)}{\left\{1-\exp\left[-1.2564\left(t/t_{m}\right)\right]\right\}}^{2}\;(\text{instantaneous nucleation})$$

(2) $${\left(I/I_{m}\right)}^{2}=\frac{1.2254}{\left(t/t_{m}\right)}{\left\{1-\exp\left[-2.3367{\left(t/t_{m}\right)}^{2}\right]\right\}}^{2}\;(\text{progressive nucleation})$$

From the non-dimensional (*I/I_m_*)^2^~(*t/t_m_*) curves of both coatings, it is observed that the nucleation model of the Ni–Co alloy and the Ni–Co–Y_2_O_3_ composite coatings follows the instantaneous nucleation model, the nucleation mechanism of the Ni–Co deposits is not changed by the incorporation of nano-Y_2_O_3_ particles with a metal matrix. However, when *t/t_m_* > 1, the experimental (*I/I_m_*)^2^~(*t/t_m_*) curves gradually deviate from the theoretical curves. This is because the theoretical model is based on the nucleation/growth occurring on a smooth cathode surface; however, there may be several dislocations and scratches on the actual electrode surface, which provide additional active nucleation sites for the electro-crystallization of metal ions. The decay rate of the current versus time was lower than the theoretical value and, accordingly, the (*I/I_m_*)^2^ was higher than the theoretical value.

On the other hand, the possible reduction process of Ni^2+^ and Co^2+^ in acidic sulfamate electrolyte solution is given by Reference [9,25,26]:

(3) $$H_{3}O^{+}+2e^{-}\to H_{2}+OH^{-}$$

(4) $$M^{2+}+e^{-}+OH^{-}\to M{\left(OH\right)}_{ads}$$

(5) $$M{\left(OH\right)}_{ads}+e^{-}\to M+OH^{-}$$

where *M* is Ni and Co, M^2+^ is Ni^2+^ and Co^2+^. It is widely accepted that the electrochemical deposition of Me^2+^ (Me = metal) takes place in several steps, and most authors accept that an intermediate Me^+^ adsorbed on the cathode surface is formed during the cathodic reaction of Ni^2+^ and Co^2+^ in an acidic electrolyte bath. The M^2+^ firstly obtains an electron and combines with OH^-^ to form the intermediates (*M(OH)_ads_*). Next, the fresh deposit layer is fabricated by the *M(OH)_ads_* adsorbed on the cathode surface, obtaining a further electron. During the electrodeposition process, the concentration of metal ions on the cathode surface maintains a steady value. However, the metal ions’ concentration on the surface of the fresh matrix layer is less than that in the electrolyte solution [26], and there is concentration polarization on the cathode surface. Meanwhile, according to Equation (3), the hydrogen evolution reaction is accompanied by nucleation/growth progress of the metal ions in aqueous solution, which provides an additional cathodic current density for the entire electrodeposition process. All these factors might result in the deviation between the experimental and theoretical curves, and therefore the nucleation/growth of the metal matrix and the hydrogen evolution should be considered together in CA curves. Palomar-Pardave et al. [27] have proved this and proposed a nucleation/growth model of metal ions to account for the overall current density and to calculate the kinetic parameters in electrodeposition, which is given in Equation (6):

(6) $$i(t)=\left\{Z_{PR}FK_{PR}{\left(\frac{2c_{0}M}{\pi \rho }\right)}^{\frac{1}{2}}+\left(\frac{2FD^{\frac{1}{2}}c_{0}}{\pi^{\frac{1}{2}}}\right)t^{-\frac{1}{2}}\right\}\times \left\{1-\exp\left[-N_{0}\pi {\left(\frac{8\pi c}{\rho }\right)}^{\frac{1}{2}}D\left(t-\frac{1-\exp\left(-At\right)}{A}\right)\right]\right\}$$

where *c*_0_ is the concentration of the metal ions in the bulk of the solution; *F* is the Faraday constant (C·mol^−1^); *Z_PR_F* is the molar charge (C·mol^−1^) in hydrogen ion reduction; *K_PR_* is the rate constant of hydrogen evolution reaction (mol·cm^-2^·s^−1^); A is nucleation rate (s^−1^); *N*_0_ is the largest nuclear number density or surface active site number (cm^−2^); other parameters also indicate its common significance. In this equation, *i* and *t* have a functional relationship to calculate kinetic parameters in electrodeposition through non-linear fitting of the experimental data (using the Marquardt–Levenberg algorithm). Equation (6) was simplified, where *P*_1_^*^ = *Z_PR_FK_PR_*(2*c*_0_*M*/π*ρ*)^1/2^, *P*_2_ = *N*_0_π*kD*, *k* = (8π*c*_0_/*ρ*)^1/2^, *P*_3_ = *A*, and *P*_4_ = 2*FD*^1/2^*c*_0_/π^1/2^. The parameters, including P_1_^*^, P_2_, P_3_, and P_4_ can vary freely in the non-linear fitting calculation process [27].

**Figure 7:**
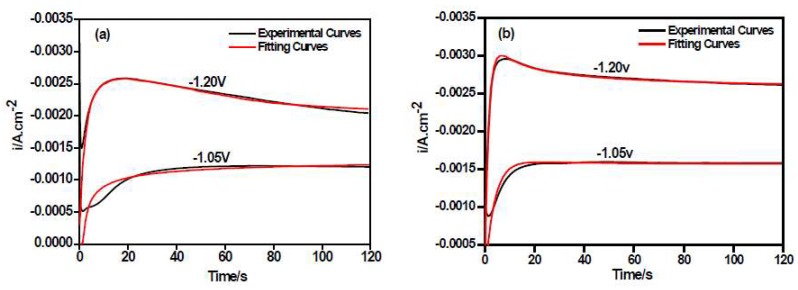
Non-linear fitting of experimental transient curves by Equation (6): (**a**) Ni–Co alloy; (**b**) Ni–Co–Y_2_O_3_ composite, ultrasonic power 100 W, T = 40 °C, and pH = 4.

Figure 7a,b shows the comparison between the theoretical I~t curves by the non-linear fitting calculation and the experimental data of the Ni–Co alloy and the Ni–Co–Y_2_O_3_ composite coatings measured under different step potentials. The fitting degree of the theoretical and experimental curves was high, and the correlation between the experimental curves and the calculated data was favorable to compute electrodeposition parameters, such as *N*_0_, the nucleation rate (*P*_3_). These calculated data for the theoretical curves coincide with the experimental results of Figure 5, (data also shown in Table 2), all calculated parameter values have four digits, with two decimal places, and the error is within 0.01. It can be observed that both the nucleation rate (*A*) and active nucleation site (*N*_0_) values of the Ni–Co–Y_2_O_3_ composite coatings are higher, from which it may be inferred that the adsorption of nano-Y_2_O_3_ particles on the cathode surface provide additional active nucleation sites for the electrodeposition of Ni^2+^ and Co^2+^ in the electrolyte, promoting the nucleation of the Ni–Co matrix. Moreover, in Figure 4a,b, the higher current densities observed in the CA curves of composite measured at more positive potential than −1.20 V (vs. SCE) can be attributed to its higher nucleation rate in this case. This is consistent with the LSV curves in Figure 2 and the *P*_3_ value in Table 2. 

**Figure 8:**
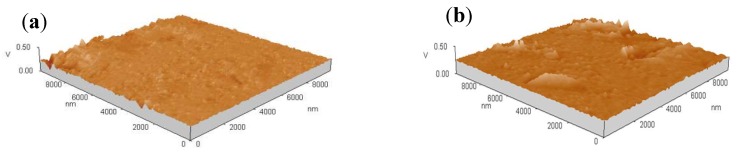

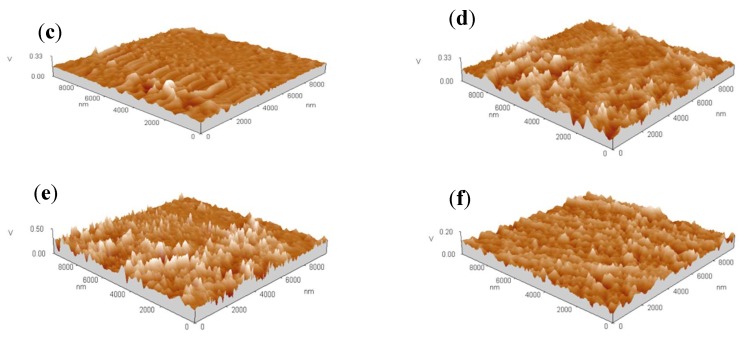
AFM images of both coatings deposited at −1.20 V (vs. SCE) for different time: (**a**) Ni–Co 5 s; (**b**) Ni–Co–Y_2_O_3_ 5 s; (**c**) Ni–Co 20 s; (**d**) Ni–Co–Y_2_O_3_ 20 s; (**e**) Ni–Co 60 s; (**f**) Ni–Co–Y_2_O_3_ 60 s.

The degree of fit between alloy and composite deposited at −1.20 V (vs. SCE) is good. To study the morphology of deposits on the substrate surface at the initial stage of electro-crystallization, shown in Figure 8, the AFM pictures observations were performed at the central area of the both coatings electrodeposited at −1.20 V(vs. SCE) for different deposition time. From Figure 8a,b, there are very few nuclei on the copper surface for both coatings, which indicates that nucleation/growth of metals ions does not take place after electrodeposition for 5 s. It is identical to the t_m_ corresponding to the experimental curves measured at −1.20 V (vs. SCE), as seen in Table 1. When the electrodeposition time is 20 s, the nuclei growing on the composite coatings are more numerous than that of alloy (Figure 8c,d). After 60 s, the copper plate is uniformly covered with the fresh layers for both deposits (Figure 8e,f). Compared with the alloy, there are more fine nuclei for the composite on the copper surface, which can be related to its high nucleation rate (*A*) and a greater number of active nucleation sites (*N*_0_). Moreover, for both coatings, the AFM images can offer favorable support for the calculated value of nucleation active sites (*N*_0_) and nucleation rate (*A*) in Table 2. 

### 3.3. Electrochemical Impedance Spectroscopy (EIS) Studies

**Figure 9:**
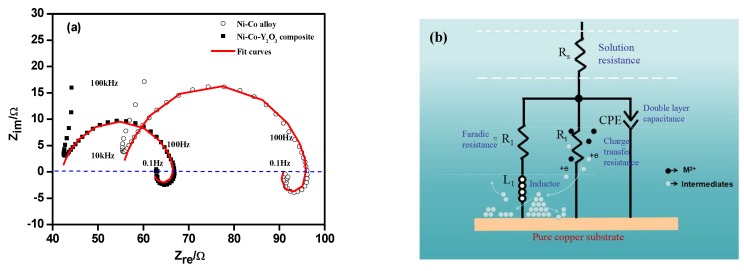
(**a**) Experimental Nyquist plots and fitting curves of coatings measured at −1.20 V (vs. SCE), ultrasonic power 100 W, T = 40 °C, and pH 4; (**b**) Simulated impedance spectra of deposits.

Figure 9a shows the experimental Nyquist plots and the fitted curves (by ZVIW 3.1) of Ni–Co deposited at −1.20 V (vs. SCE) with and without nano-Y_2_O_3_ particles in the electrolyte. The degree of correlation degree between the fitted plots and experimental curves is acceptable, and this calculated data can provide an accurate reference for electrochemical processes. In Nyquist curves, an incomplete capacitive-resistance response consists of some scattered points first appeared at high frequency (10^5^ Hz~10^4^ Hz), which may be due to the limitation of device. Both curves contained a complete capacitive loop at high frequency (10^4^ Hz~10 Hz) and an inductive loop at low frequency (10 Hz~10^−1^ Hz), the capacitive arc is related to solution resistance and the double-layer electric capacity parallel with charge transfer resistance. The inductive arc is caused by the sorption and desorption of deposit layers on cathode surface [28]. The capacitance arc radius of the Ni–Co–Y_2_O_3_ composites was smaller than with the Ni–Co alloy, indicating that the charge transfer resistance of the composite was lower. The inductive arc radius of the composite coating was slightly larger than for the alloy, showing that the formed intermediates correspond to different time constants in electrodeposition, and the nano-Y_2_O_3_ particles adsorbed on the electrode surface influenced the electrodeposition of Ni^2+^ and Co^2+^. 

Figure 9b is an equivalent circuit diagram of alloy and composite deposited at −1.20 V (vs. SCE). In this diagram, *R_s_* indicates solution resistance, *R_t_* expresses charge transfer resistance, a constant phase original CPE is used to represent a double layer capacitance, and *R_1_* is a Faraday resistor in series with inductance *L_1_*. The computational data is shown in Table 3. The charge transfer resistance of the composite was significantly less than for the alloy, which may be related to the weak adsorption of nano-Y_2_O_3_ particles on the electrode surface, increasing the active reactive area and accelerating the mass transfer rate of Ni^2+^ and Co^2+^ to cathode, thus decreasing the charge transfer resistance. This trend is similar to co-deposition of Ni with Al_2_O_3_ [19], ZrO_2_ [29], TiO_2_ [30]. Moreover, the values of R_1_ and L_1_ between alloy and composite is different, proving that the intermediate corresponding vary time constants in electrochemical deposition. The fitting data calculated are consistent with the theoretical analysis of Nyquist spectrum.

### 3.4. Microstructure of Coatings

**Figure 10:**
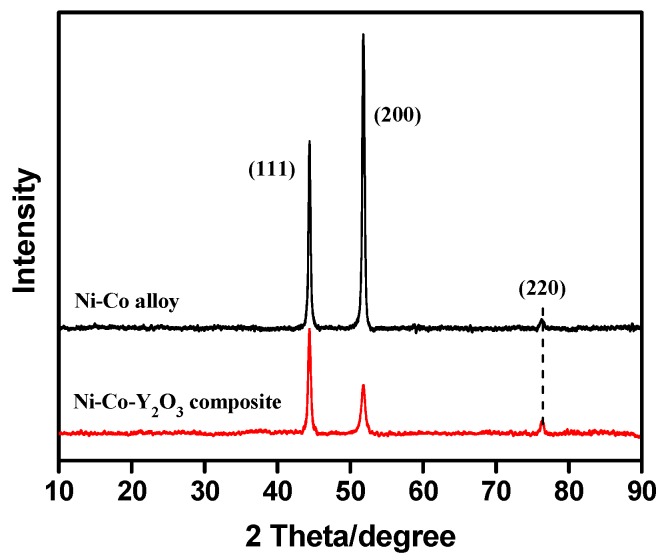
X-ray diffraction curves of Ni–Co alloy and Ni–Co–Y_2_O_3_ composite coatings.

The X-ray diffraction patterns of Ni–Co and Ni–Co–Y_2_O_3_ deposits are shown in Figure 10. For both coatings, there are three obvious diffraction peaks at 44°, 52°, and 76°, and the corresponding diffraction surfaces are (111), (200), and (220), respectively. However, compared to the Ni–Co alloy, the preferred orientation of the composite coating shifts to (111) diffraction surface. The grain size of coatings can be calculated by the Scherrer equation [31] (D = 0.9 λ/β cos θ, where λ is the diffraction wavelength (0.15405 nm), β is the half-width of the diffraction peak, and θ is diffraction angle of the peak). The calculated data show that the average grain size of the Ni–Co layer was 43.7 nm, and the average grain size of the Ni–Co–Y_2_O_3_ composite coating was 38.7 nm.

**Figure 11:**
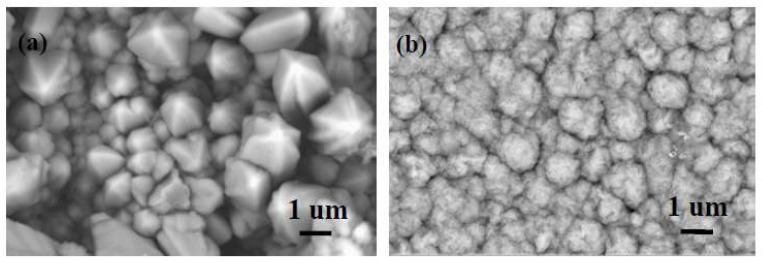
Scanning electron microscope (SEM) images for the coatings: (**a**) Ni–Co alloy; (**b**) Ni–Co–Y_2_O_3_ composite coating.

Figure 11 shows the surface morphology of the Ni–Co alloy and the Ni–Co–Y_2_O_3_ composite coatings. The atomic percentage of each element in the coatings, as checked by EDS, is shown in Table 4. Figure 11a shows incoherent and globular surface morphology, with bulges clearly visible, which is related to the non-uniform nucleation and lower nucleation rate shown in Table 2. In Figure 11b, the surface of the composite is formed with a larger number of finer globular grains. The main reason is that the presence of nano-Y_2_O_3_ influences the competition of metal nuclei and crystal growth, which means that more nucleation sites are available for the metal ions and crystalline growth is suppressed during electrodeposition. Consequently, the composite coating is more uniform and compact, with a finely grained morphology.

## 4. Conclusions

Ni–Co alloy and Ni–Co–Y_2_O_3_ composite coatings are successfully fabricated by ultrasound-assisted electrodeposition from an acid sulfamate electrolyte bath. Linear sweep voltammetry (LSV) curves show that the addition of nano-Y_2_O_3_ particles in the electrolyte reduces the deposition overpotential. The CA curves indicate that the nucleation rate increased when the transient potentials are more negative, and the addition of nano-Y_2_O_3_ particles accelerates the nucleation process of Ni^2+^ and Co^2+^. The fit data calculated are in good agreement with the experimental curves. The AFM images of both coatings deposited for different periods of time support the fit data calculated. The EIS test shows that the charge transfer resistance of composite is lower than for alloy, and an equivalent circuit diagram and calculated data (by ZVIEW 3.1) can offer a favorable reference for the EIS curves. The incorporation of nano-Y_2_O_3_ particles in the matrix changes the preferred orientation and produces a more uniform and compact deposit layer with finely grained microstructure.

## Figures and Tables

**Table 1:** *I_m_* and *t_m_* of I~t curves for alloy and composite deposited at −1.05 V (vs. SCE) to −1.20 V (vs. SCE).

Potential/V (vs. SCE)	Ni–Co Alloy		Ni–Co–Y_2_O_3_	
	*I_m_* (A·cm^−2^)	*t_m_* (s)	I_m_ (A·cm^−2^)	*t_m_* (s)
−1.05 V	−0.00122	76.1	−0.00160	49.2
−1.10 V	−0.00169	31.8	−0.00209	22.5
−1.15 V	−0.00197	21.9	−0.00256	12.3
−1.20 V	−0.00258	18.6	−0.00296	8.5

**Table 2:** Optimal nucleation kinetic parameters derived from Equation (5).

Materials	Potential	*P*_1_^*^ (μA cm^−2^)	*P*_2_ (s^−1^)	*P*_3_ (s^−1^)	*P*_4_ (μA cm^−2^)	*A* (s^−1^)	*N*_0_ × 10^6^ (cm^−2^)
Ni–Co	−1.05 V	−1.37	0.14	0.75	−1.52	0.75	3.92
Ni–Co	−1.20 V	−2.07	0.33	1.98	−1.59	1.98	5.47
Ni–Co–Y_2_O_3_	−1.05 V	−1.55	0.26	1.28	−1.54	1.28	4.61
Ni–Co–Y_2_O_3_	−1.20 V	−2.48	0.54	2.39	−1.55	2.39	6.38

**Table 3:** Impedance parameters of materials electrodeposition at −1.20 V.

Materials	Potential (V)	*R_s_*/Ω·cm^2^	CPE_1_-P/F·cm^2^	*R_t_*/Ω·cm^2^	*R_1_*/Ω·cm^2^	*L_1_*/H·cm^2^
Ni–Co	−1.20	55.21	5.09 × 10^-5^	41.48	221.9	15.61
Ni–Co–Y_2_O_3_	−1.20	51.92	4.98 × 10^-5^	25.29	106.8	4.72

**Table 4:** Atomic percentage of each element in the coatings (wt. %).

Materials	Ni	Co	O	Y	Total
Ni–Co	67.32	32.68	–	–	100
Ni–Co–Y_2_O_3_	59.55	20.69	14.11	5.65	100
